# Integration of experimental data and use of automated fitting methods in developing protein force fields

**DOI:** 10.1038/s42004-022-00653-z

**Published:** 2022-03-18

**Authors:** Marcelo D. Polêto, Justin A. Lemkul

**Affiliations:** 1 Department of Biochemistry, Virginia Tech, Blacksburg, VA 24061 United States; 2 Center for Drug Discovery, Virginia Tech, Blacksburg, VA 24061 United States

**Keywords:** Biophysical chemistry, Molecular dynamics

## Abstract

The development of accurate protein force fields has been the cornerstone of molecular simulations for the past 50 years. During this period, many lessons have been learned regarding the use of experimental target data and parameter fitting procedures. Here, we review recent advances in protein force field development. We discuss the recent emergence of polarizable force fields and the role of electronic polarization and areas in which additive force fields fall short. The use of automated fitting methods and the inclusion of additional experimental solution data during parametrization is discussed as a means to highlight possible routes to improve the accuracy of force fields even further.

## Abstract (web summary)

Molecular dynamics simulations, used to study chemical and biophysical processes, rely on the accuracy of the employed force fields. Here, the authors review successes and key areas of difficulty in the development of additive and polarizable force fields, and discuss experimental data availability, how empirical refinement impacts parametrization, and highlight possible routes to further improve the accuracy of force fields.

## Introduction

Molecular dynamics (MD) simulations have been widely used to study chemical and biophysical processes with atomistic resolution^1,2^. These simulations rely on the accuracy of the so-called force fields (FFs), mathematical functional forms, and the associated parameters that describe the relationship between a given set of atomic coordinates, $\overrightarrow{R}$, and its potential energy, *U*($\overrightarrow{R}$).

The quality of any force field is assessed by its ability to reproduce structural, dynamic, and thermodynamic properties of a system, given enough sampling. Developing an accurate FF is a complex task, as deriving parameter sets that can accurately reproduce experimental data is challenging and time-consuming. The functional form underlying a FF is composed of multiple expressions associated with different energy terms, each one describing specific types of interactions between atoms. A common functional form is shown in Eq. (1):

1 $$U(\overrightarrow{R})=	\mathop{\sum}\limits_{{{{{{\mathrm{Bonds}}}}}}}{k}_{b}{(b-{b}_{0})}^{2}+\mathop{\sum}\limits_{{{{{{\mathrm{Angles}}}}}}}{k}_{\theta }{(\theta -{\theta }_{0})}^{2}+\mathop{\sum}\limits_{{{{{{\mathrm{Dihedrals}}}}}}}{k}_{\phi }[1+cos(n\phi -\delta )]\\ 	 +\mathop{\sum}\limits_{LJ}4{\varepsilon }_{ij}\left[{\left(\frac{{\sigma }_{ij}}{{r}_{ij}}\right)}^{12}-{\left(\frac{{\sigma }_{ij}}{{r}_{ij}}\right)}^{6}\right]+\mathop{\sum}\limits_{{{{{{\mathrm{Coulomb}}}}}}}\frac{{q}_{i}{q}_{j}}{4\pi {\varepsilon }_{0}{r}_{ij}}$$

In Eq. (1), *b* is the bond length, *θ* is the valence angle, *ϕ* is the dihedral angle. The bond force constant and equilibrium distance are *k*_*b*_ and *b*_0_, angle force constant and equilibrium angle are *k*_*θ*_ and *θ*_0_, the dihedral force constant, multiplicity and phase angle are *k*_*ϕ*_, *n* and *δ*, respectively. Together, these terms are referred as bonded terms. The nonbonded terms between atoms *i* and *j* are composed of electrostatic interactions between their respective partial charges *q*_*i*_ and *q*_*j*_ separated by a distance *r*_*ij*_ and by van der Waals interactions, modeled by a 12-6 Lennard-Jones (LJ) function using an LJ well-depth *ε*_*ij*_ and a radius *σ*_*ij*_, which defines the distance at which the interatomic LJ potential is zero.

Over the years, many different FFs have been developed and refined, including the well-known CHARMM^3–8^, AMBER^9–14^, OPLS^15–19^, and GROMOS^20–24^ families. While the first generation of protein FFs was developed with the primary goal of maintaining the tertiary structure of folded proteins^9,25^, later generations have been refined using more robust and diverse experimental data, as well as more accurate quantum mechanical (QM) calculations, which have improved FF robustness and accuracy.

The most widely used FFs rely on fixed, atom-centered charges, such that the interaction energy in a system is the sum of all the pairwise interactions. For this reason, such FFs are classified as additive FFs; that is, the removal of any set of atoms from a system does not affect the interaction energies among the remaining atoms because there are no multibody effects. Recently, new functional forms that contain explicit terms to model the change in the electronic structure of a molecule in response to alterations of the local electric field have emerged. These polarizable FFs are non-additive; the removal of any atoms impacts the inducible dipoles in the system, leading to different interaction energies among the remaining atoms.

Here, we review the most common strategies used in the development of additive and polarizable force fields for proteins, the relationship between experimental data used as a reference, biases in force field development and automated methods that may overcome them, and the challenge of modeling structural, dynamic, and thermodynamic properties accurately in multiple chemical environments using additive and polarizable force fields.

## Force field development strategies

Multiple parametrization strategies have been adopted by each FF family over the years, using different types of experimental data for calibration/validation, as well as different methods (automated and empirical) to derive parameters sets. In this section, we review general strategies applied in FF parametrization and highlight novel strategies that have recently been applied in FF refinement.

### Bonded terms

Bond and angle parameters are the core basis of a protein FF and are usually modeled via harmonic functions, which are fit to spectroscopic data or crystal geometries. When necessary, QM calculations can be employed to obtain optimized geometries or vibrational frequencies as well, from which equilibrium bond and angle values can be derived. Although these parameters are kept mostly untouched throughout FF generations, recent work by König and Riniker^26^ suggested that they could be tuned to better capture bond and angle responses to changes in the local environment^27^, approximating the observed QM behavior and yielding a better representation of the potential energy landscape.

With respect to bonded terms, the major challenge in describing protein structure and dynamics is related to torsional parameters. Dihedral terms are used to correct inaccurate torsional preferences that arise from the simplification of using Coulomb and LJ potentials to model nonbonded interactions between atoms separated by three bonds. The molecular mechanics (MM) approximation underlying common FFs lacks orbital effects and conformation-dependent polarization response that are important at this length scale. Therefore, cosine potentials are applied to torsions to recover the correct configurational energy profile as a function of dihedral rotation, thus yielding conformational populations. In the derivation of protein FFs, careful attention must be paid to backbone dihedrals (particularly *ϕ* and *ψ*), which govern secondary structure stability, and sidechain dihedrals, which dictate rotamer preference, with *χ*_1_ also being linked to the two key backbone dihedrals.

Early force fields developed dihedral parameters by targeting a small number of low-energy conformations of dipeptides in gas-phase via QM calculations, with special attention given to *ϕ*/*ψ* backbone torsions^15,28^. Further refinements focused on performing more robust QM calculations to scan potential energy surfaces (PES) for backbone *ϕ*/*ψ* torsions^4,13,16,17,29,30^ or sidechain *χ*_*n*_ torsions^12,13,16,17,31^ in dipeptides. These efforts improved agreement with experimental ^3^J-coupling constants and crystallographic sidechain rotamer distributions. However, the inclusion of more robust ab initio calculations was not enough to reproduce experimentally observed backbone conformational preferences, requiring empirical adjustments. The main limitation in this approach was proposed to be the use of molecular fragments (usually capped amino acids or small peptides), which might not necessarily capture the cooperativity in conformational sampling observed in polypeptides and proteins.

To account for the coupled dynamics of *ϕ*/*ψ* torsions and their subsequent conformational preferences, MacKerell et al. ^4^ introduced a 2D correction map (CMAP) in the CHARMM22/CMAP force field. A CMAP term is included in the functional form as an additional energy term that is the difference between QM and MM energies over the entire 2D *ϕ*/*ψ* conformational space. The authors studied capped dipeptides in a vacuum to obtain CMAPs for proline, glycine, and a general correction based on alanine, which was applied to all other amino acids. Applying such CMAPs made it possible to reproduce 2D QM energy surfaces, but led to deviations in *α*-helical and *β*-sheet structure when simulating proteins, thus requiring empirical modifications to recover agreement with experimental data. MacKerell et al. ^4^ concluded that the polarization response in the gas phase and, consequently, the PES obtained from QM calculations in vacuum are different from the ones obtained in solution. Later refinements in CMAPs were presented in CHARMM36^6^ and CHARMM36m^8^ using better ab initio methods (RI-MP2/cc-pVT(Q)Z) and more robust empirical adjustments based on PDB surveys or NMR structural data. Thus, it is clear that even when targeting high-level QM data, it is challenging to generate suitable FF parameters, which still rely on the use of empirical data on protein conformational ensembles to produce the final parameters.

The development of the AMBER ff15-FB^31^ is a demonstration of such a challenge. The authors employed the ForceBalance algorithm^32^, an automatic optimization method that uses experimental and QM target data to fit multiple parameters at once. In ff15-FB, bond, angle, and dihedral parameters from the ff99SB parameter set were refined by ForceBalance by targeting RI-MP2/aug-cc-pVTZ gas-phase QM calculations of *ϕ*/*ψ* 2D potential energy and vibrational frequencies. Although nonbonded parameters were not modified, the authors were able to better reproduce S^2^ order parameters, NMR scalar couplings, and temperature dependence of secondary structure. However, the authors stressed that even though ForceBalance was able to accurately fit *N*-dimensional parameters at once, targeting gas-phase QM data proved to be a bottleneck when fitting parameters in conjunction with condensed-phase data.

Subsequently, Cerutti et al. ^33^ proposed a solution to this inconsistency of deriving parameters for use in the condensed phase but targeting gas-phase data by introducing a modification of the AMBER force field, named IPolQ model, which implicitly accounts for polarization effects. In its first version (the ff14ipq FF), partial charges are implicitly polarized to improve the balance between solute–solvent and solute–solute interactions. More information regarding nonbonded treatment in this model will be discussed in Section Nonbonded terms. Briefly, partial charges of an amino acid are predicted to be halfway between the QM charges of a dipeptide in a vacuum and in the presence of a reaction-field potential that models water. This approach allowed the authors to implicitly account for polarization effects when targeting QM PES obtained in a vacuum to derive dihedral parameters. Overall, ff14ipq showed good agreement with *ϕ*/*ψ* distributions of model peptides and yielded generally stable protein dynamics. Nevertheless, refinements were presented by Debiec et al. ^34^ in an updated FF version, ff15ipq. More specifically, new atom types were introduced for backbone atoms, allowing for more specific dihedral refinements. Validation simulations were carried out on globular proteins, short peptides, and intrinsically disordered proteins (IDPs). ff15ipq produced good agreement with challenging experimental data, such as the temperature-dependent unfolding of K19 and (AAQAA)_3_ model peptides or folding events of IDPs upon binding. Overall, the results from the IPolQ model emphasize the importance of developing force fields in a physically consistent manner, especially when deriving torsional parameters.

Following the CMAP logic applied to CHARMM force fields, the Simmerling group used ff14SB^13^ as a starting point to developed residue-specific CMAPs for the AMBER FF family, leading to ff19SB^14^. Tian et al. developed CMAPs for 16 residues that are applied to all 20 natural amino acids. While the CHARMM CMAP philosophy uses alanine CMAP as a general correction to other amino acids, ff19SB uses leucine CMAP to correct phenylalanine, tryptophan, tyrosine, cysteine in disulfide bonds and all three protonation states of histidine. The remaining amino acids have specific corrections, including different protonation states for aspartate and glutamate. Another difference from the CHARMM CMAP philosophy is how ff19SB deals with the polarization inconsistency of targeting gas-phase QM PES. The authors derived CMAPs by calculating the QM PES using the SMD implicit solvent model, whereas MM PES were calculated using a generalized Born (GB-Neck2) continuum solvation model. It is important to notice that while solving the polarization inconsistency when deriving dihedral parameters, the ff19SB still uses partial charges based on gas-phase QM calculations. In contrast to the CHARMM FF series, Tian et al. ^14^ were able to reproduce secondary structure preference for different amino acids without empirically adjusting CMAPs. Also, amino acid-specific NMR properties such as scalar coupling and helical propensities were in good agreement with experimental data, as well as S^2^ order parameters for folded proteins. Lastly, ff19SB could only accurately predict experimental behavior when used in combination with the OPC water model, suggesting a dependence on the water model.

Another novel approach was the recently reported environment-specific force field (ESFF1)^35^. The authors built a database containing *ϕ*/*ψ* values of tripeptide sequences extracted from structures deposited in the Protein Data Bank (PDB). Each sequence was classified based on the chemical properties of N- and C-terminal residues, either polar (P) or nonpolar (NP). This classification led to four possible configurations: P-X-P, NP-X-NP, P-X-NP, and NP-X-P, with X being each of the 20 canonical amino acids. Subsequently, the authors evaluated 80 specific environments to derive 71 backbone CMAP corrections in an attempt to implicitly account for sequence-specific conformational preferences. In this approach, parameters are assigned to a residue based on its neighboring residues during topology construction, challenging the “additivity” assumption common to all force fields. ESFF1 demonstrated a good agreement with NMR chemical shifts and ^3^J-coupling constants of tetrapeptides and short peptides. By applying replica-exchange MD simulations, the authors were able to demonstrate the folding of fast-folding proteins. Interestingly, ESFF1 was able to reproduce the structural behavior of both ordered and disordered proteins, suggesting that such an approach to deriving torsional terms may be promising in future FF development.

Overall, these strategies highlight the complexity of assigning protein torsional parameters and their dependence on the target data used. While QM calculations can be a good starting point, capturing intricate protein dynamics requires an additional step of empirical adjustment based on experimental data, typically in the form of structural surveys and NMR data such as chemical shifts and ^3^J-coupling constants. Also, the model chemistry employed in QM calculations can impact target CMAP corrections and torsional barriers, which can be a source of differences in the performance of FF families. It is also important to mention that *ϕ*/*ψ* distributions obtained from PDB surveys that are commonly used as target data are usually averages over thousands of different structures, and care must be taken when rebalancing against these geometries since some error in their assignments may be due to e.g. resolution and crystal packing effects from X-ray experiments and the empirical nature of torsional assignment with NMR. Lastly, the incorporation of solution data in the training set has the potential to improve the accurate modeling of proteins in solutions (discussed in Section More diverse structural data).

### Nonbonded terms

In the MM convention, interactions between atoms that are not covalently bonded are typically described by the sum of van der Waals and electrostatic terms. The most common representation of van der Waals interactions is the 12-6 LJ potential shown in Equation (1). The LJ potential is commonly tied to an “atom type” logic, in which each atom type is assigned *σ* and *ε* values. The same chemical element can have multiple atom types to more accurately model interactions between heterogeneous moieties, allowing the FF to cover a wide range of chemically diverse molecules. Since LJ parameters are assigned to each atom type, an LJ interaction between a pair of atom types is determined by a “combination rule” (or “mixing rule”) to model a pairwise interaction. Such rules are generally applied to all-atom types within the force field, which requires internally self-consistent calibration efforts. Due to its simple nature, combination rules may fail to accurately describe the optimal LJ interaction distance and its strength for a specific pair of atom types. In such cases, most force fields allow pair-specific values of *σ*_*ij*_ and *ε*_*ij*_ that override their combination rule. Such LJ corrections were pioneered by the CHARMM force field and are commonly referred to as “off-diagonal” or NBFIX terms.

Early LJ parameters were developed by targeting condensed-phase properties of pure liquids, molecular volumes, crystallographic data, and QM interaction energies^36–40^. More recent LJ optimization efforts have focused on refining existing LJ parameters to create new atom types or to correct existing ones, often using off-diagonal terms to correct interaction energies^8,19,23,24,34,41^.

Electrostatic interactions are usually described by Coulomb’s Law, in which each atom is assigned a partial charge and interaction strength decays with *r*^-1^ dependence. Partial charge assignment is usually based on gas-phase QM calculations of the electron distribution of a molecule, specifically targeting electrostatic surface potentials or molecular dipole moments. In additive force fields, the assigned charges in a molecule are often strategically over-polarized to account for the expected polarization effects in the aqueous phase. In doing so, additive force fields simplify the many-body components involved in electronic polarization effects to a one-body problem (i.e., a mean-field approximation) by assuming a constant dielectric medium surrounding a molecule. In reality, the electronic medium around a protein residue is frequently heterogeneous, impacting the molecular polarization response and, in turn, the respective interaction energies between the molecule and its environment. The effects of explicitly modeling electronic polarization will be discussed in Session Polarizable force fields.

Nonbonded interactions are perhaps the most challenging to parametrize and different approaches to calibrate them have been developed over the years. The AMBER FF family typically uses a rescaling factor on the partial charges obtained via gas-phase QM calculations using the HF/6-31G* model chemistry, which fortuitously over-polarizes partial charges and is used to account for polarization as explained above. Some attempts to achieve better-condensed phase behavior have been made by assuming continuum solvent models during QM calculations^42^. More recently, He et al. ^43^ developed a new semiempirical ab initio method (named ABCG2) to derive new partial charges for small molecules when using the General AMBER Force Field (GAFF) to reproduce solvation free energies of small organic molecules. In the CHARMM FF, the initial set of partial charges are obtained by targeting a ~20% higher dipole moment calculated via gas-phase MP2/6-31G* QM calculations. These charges are later validated and refined based on water interactions calculated in both MM and QM levels. In some additive FFs, like the GROMOS and OPLS families, partial charges are empirically adjusted to target condensed-phase properties such as heat of vaporization, liquid density, and free energy of solvation. By targeting properties in aqueous and nonpolar media simultaneously, such charges implicitly account for polarization responses in condensed-phase and are assumed to better reproduce interaction energies in both polar and nonpolar environments frequently found in protein simulations. Nevertheless, this approach also adopts a mean-field approximation by deriving partial charges in homogeneous media (organic liquid or water) modeled via nonpolarizable solvent molecules.

The IPolQ model^33,34^ is also novel in its attempt to implicitly represent polarization effects. In its parametrization, dipeptides were initially simulated with the AMBER ff99SB force field to obtain water distributions and configurations around chemical moieties. These water molecules were replaced by perturbing charges in QM calculations to generate a polarized electrostatic surface potential (ESP) of the dipeptide, thereby approximating the many-body component of the electronic polarization effects. In addition, a gas-phase ESP was also calculated via QM methods and the charges assigned in the IPolQ model are assumed to be the average between the charges in the polarized system and the charges in a vacuum. Thus, the IPolQ model accounts for a heterogeneous polarization response during charge assignment while still remaining nonpolarizable. Simulations using ff15ipq showed that conformational dynamics of globular proteins and disordered peptides were in good agreement with experimental data. Moreover, ff15ipq is one of the most accurate FFs in describing the probability of ion-pairing in solution among the additive FFs, emphasizing the importance of considering polarization during charge assignment.

Ultimately, all FFs aim to strike an optimal balance while reproducing solute–solute and solute–solvent interactions, which is important for accurately modeling protein dynamics in an aqueous medium. Such a balance is critical when simulating proteins encountering chemically distinct environments, like when embedded in membranes, or when describing protein–protein interaction. Doing so is particularly challenging for additive FFs due to their nonpolarizable nature, in that they are not generally calibrated to account for media of lower polarity.

Such balance has been highlighted by attempts to simulate IDPs using additive FFs; several popular FFs were shown to overestimate the secondary structure in IDPs and yield structures that were too compact^44–46^. Some corrections were proposed by tuning backbone torsional parameters, leading to the development of IDP-specific force fields, such as CHARMM36IDPSFF^47^, ff14IDPSFF^48^, and OPLSIDPSFF^49^. Other efforts aimed to correct the balance between protein–protein and protein–water interactions via LJ corrections or the creation of new atom types to specifically describe conformational dynamics of IDPs^8,35,41,50^. All of these refinements emphasize the difficulty in simultaneously describing intramolecular and intermolecular properties with sufficient accuracy.

Modifying protein–water interactions has been frequently attempted in an effort to better represent the dynamics of difficult systems like IDPs or other unfolded/partially folded states of proteins and polypeptides. The AMBER ff99SB-disp^41^ used an analogous approach: the authors tuned the FF to be compatible with the TIP4P-D water model, which was developed to model water dispersion interactions more accurately^51^. Robustelli et al. adjusted LJ terms and backbone torsional parameters to better balance intra- and intermolecular interactions. By doing so, the authors were able to produce less compact structures of disordered proteins while still preserving the conformational dynamics of folded proteins, making it possible to use ff99SB-disp to simultaneously study both folded and unfolded proteins. These results suggest that modeling solute–solute and solute–solvent interactions using additive force fields requires some sort of compromise in nonbonded parameter refinement.

Although these approaches have been generating remarkable results, additive force fields assume that solute–solvent and solute–solute interaction energies are insensitive to the electronic medium surrounding the molecules. For instance, it is quite common to observe changes in the chemical environment of protein residues during an MD trajectory, like the increase in solvent accessibility of buried residues or vice-versa. The change in the electronic properties of the media surrounding protein residues triggers an electronic response in the molecules, which in turn impacts the solute–solvent and solute–solute interaction energies. In additive force fields, such responses are not taken into account, thus challenging the development of parameters that can simultaneously describe a wide range of electronic and conformational events in protein conformational dynamics.

### Polarizable force fields

When it comes to macromolecular force fields, reproduction of structural data is of critical importance, but also correct energies are required to properly balance intra- and intermolecular interactions. Obtaining such a balance is directly linked to correct thermodynamics and kinetics (e.g., correct balance in conformational ensemble and accurate conformational transition rates). Therefore, a model that explicitly accounts for polarization effects has the potential to better reproduce interaction energies in different chemical environments^52–55^.

Overall, polarizable force fields are usually based on a variation of the functional form presented in Equation (1), augmented by an explicit term to model electronic degrees of freedom. Currently, the most extensive polarizable force fields are the AMOEBA force field^55–57^ and the Drude force field^52,53,58^.

#### AMOEBA force field

The AMOEBA FF is classified as an induced dipole and multipole model. It extends the partial charge representation used in Equation (1) to incorporate atomic multipoles, which include monopoles (partial charges), dipole vectors, and quadrupole tensors. Together, the interactions between the atomic multipoles describe the so-called permanent electrostatic interactions. In addition, an explicit polarization term models the electronic polarization dynamically via a self-consistent relaxation scheme, in which the atomic dipoles of all atoms are subject to the electric field exerted by permanent electrostatics and induced dipoles. Additionally, the AMOEBA FF uses a damping function proposed by Thole^59^ to “smear” the atomic multipoles and avoid over-polarization via the expression below:

2 $$\rho =\frac{3a}{4\pi }exp\left[-a{\left(\frac{{r}_{ij}}{{({\alpha }_{i}{\alpha }_{j})}^{1/6}}\right)}^{3}\right]$$

in which *a* determines the strength of the damping and *α* is the atomic polarizability of each atom participating in the interaction. In practice, both *α* and the Thole factor *a* become new parameters to be fit alongside the atomic multipoles when calibrating the FF.

The first version of the AMOEBA protein FF (AMOEBA13) was developed by Shi et al. ^55^. Remarkably, AMOEBA13 reproduced QM dipole moments of dipeptides and tetrapeptides in a near-perfect agreement. Torsional parameters for *ϕ* and *ψ* were fit against gas-phase 2D QM potential energy surfaces using alanine, proline, and glycine dipeptides. In contrast to the CMAP approach described above, the difference between QM and MM energies were used to derive cosine parameters for *ϕ* and *ψ*. It is important to note that polarizable FFs do not suffer from the inconsistency of deriving parameters in the gas phase to be used in the condensed phase due to the explicit modeling of polarization response while deriving those MM parameters.

Torsional parameters were later refined against *ϕ* and *ψ* distributions in the PDB by assigning weighting factors to conformers in the polyproline (PPII), *α*-helical and *β*-sheet regions. This approach yielded a good agreement with the J-coupling values of Ala_5_. Without refining with empirical data obtained from the PDB, the authors obtained worse structural agreement, demonstrating the importance of combining experimental structural data and robust QM calculations when deriving torsional parameters. Moreover, the authors evaluated ten well-studied folded proteins for 30 ns, producing RMSD values of ~1 Å. When compared with experimental J-coupling NMR data, AMOEBA13 produced results comparable to AMBER additive ff99SB and ff99SB-ildn FFs, a remarkable outcome, considering that AMOEBA was not parametrized against NMR data. Although NMR data also suggest that protein loops in AMOEBA13 are somewhat too flexible, the overall agreement was good.

Following AMOEBA13, the electrostatic treatment used in the functional form was revised by Rackers et al. ^60^, who proposed an optimized charge penetration model for the AMOEBA force field. Later, Das et al. ^61^ revisited the atomic polarizability implemented in AMOEBA13 to propose the inclusion of anisotropic polarizability to better reproduce QM interaction energies. The authors demonstrated the importance of anisotropy by calculating interaction energies in clusters of water molecules, which were subsequently more accurate.

The AMOEBA18 FF was later released and included parameters for nucleic acids and an improvement in atomic multipoles for proteins^57^. This new FF was used by Célerse et al. ^62^ to study the role of electronic polarization in the dissociation of protein–protein complexes and unfolding of peptides. By simulating the unfolding dynamics of Ala_10_ in a vacuum using both AMOEBA13, AMOEBA18, and additive FFs, the authors showed that the AMOEBA FFs produced free-energy profiles with much lower barriers due to the polarization response to the gas phase. Another test was the dissociation of protein–protein complexes, in which the AMOEBA FFs were able to better reproduce protein–protein dissociation enthalpy due to the better description of salt bridge energies and dynamics. Such thermodynamic properties, e.g., unfolding and dissociation free energies, are therefore useful and rigorous tests of FF quality.

Another feature in which better electrostatic description should play an important role in the study of electric field organization within enzyme active sites. In this regard, the studies involving ketosteroid isomerase (KSI) are perhaps the most well documented^63–68^. The Boxer group studied the electric field magnitude and directionality exerted by the KSI active site onto the C=O bond of substrates using the additive ff99-ILDN FF, observing a near-perfect agreement with the experimental electric field^65^. Interestingly, similar results were obtained by Wang et al. ^69^ using ab initio path integral MD simulations and by Welborn and Head-Gordon^67^ using the AMOEBA polarizable force field, although the magnitude of the field in the latter study was slightly lower. As reported by Wang et al. ^69^, the KSI electric field is strongly dependent on the precise chemical positioning of active site residues, such as Tyr16 and Asp103 that together contribute to the vast majority of the total field. These findings are supported by Fried et al. ^65^, who observed that electric field magnitudes could decrease by ~50% if the hydrogen-bond network stabilizing the substrate pose was disrupted. Such results suggest that, given the correct configuration, ff99-ILDN FF was sufficiently accurate to reproduce the field magnitude exerted by KSI active site.

More recently, Bradshaw et al. ^70^ studied the electric field exerted by peptidyl-prolyl isomerase cyclophilin A (CypA) in its active site using AMOEBA13, AMBER ff14SB, and CHARMM36m FFs. In contrast to the findings for KSI, Bradshaw et al. ^70^ demonstrated that both additive FFs substantially overestimated the electric field in the CypA active site, while AMOEBA produced near QM-level accuracy. However, it is important to mention that the authors did not compare the active site structural organization modeled by additive FFs and AMOEBA, which might have been a source of difference. Although the electric field organization of more systems must be investigated to draw an overall conclusion, the findings reported so far indicate that: (1) electric field calculations might require polarizable models to achieve quantitative accuracy and (2) they are very dependent on precise chemical positioning of protein atoms.

#### Drude force field

Another strategy to model electronic polarization was proposed by Drude et al. ^71^, in which the changes in the atomic electronic cloud are modeled via auxiliary particles attached to their parent atoms via harmonic springs. This approach was used to develop the Drude polarizable FF^52,53,58,72^. The displacement of these auxiliary Drude particles from their parent atoms is induced by the surrounding electric field, creating a charge distribution that can be calibrated to describe atomic polarization. In the Drude FF, the Drude particles are negatively charged by convention and the magnitude of the charge is assigned according to the atomic polarizability of its parent atom:

3 $$\alpha =\frac{{q}_{{{{{{\mathrm{D}}}}}}}^{2}}{{K}_{{{{{{\mathrm{D}}}}}}}}$$

in which *q*_*D*_ is the charge on the Drude particle and *K*_*D*_ is the bond force constant between the Drude particle and its parent atom. By modeling the electronic polarizability via an auxiliary particle, the Drude FF can model the relaxation of electronic degrees of freedom dynamically via extended Lagrangian integration^73^, therefore improving simulation speed over self-consistent field approaches. In contrast with additive FFs, which typically exclude nonbonded interactions between first and second bonded neighbors, vicinal dipole-dipole interactions (1–2 and 1–3 interactions) explicitly contribute to the energy function in the Drude FF. Due to the short distance, electrostatic interactions are damped via an alternative screening function proposed by Thole:

4 $${S}_{ij}({r}_{ij})=1-\left[1+\frac{a{r}_{ij}}{2{({\alpha }_{i}{\alpha }_{j})}^{1/6}}\right]{{{{{\mathrm{exp}}}}}}\left[\frac{-a{r}_{ij}}{2{({\alpha }_{i}{\alpha }_{j})}^{1/6}}\right]$$

in which *a* is the Thole factor describing the damping strength. The usage of a screening function to compute 1–2 and 1–3 interactions allows a better description of molecular polarizability.

Similar to the AMOEBA FF, both *α* and Thole factors are tunable parameters in the Drude FF. Initial values of *α* are taken from atomic polarizabilities proposed by Miller^74^, while a default value of 2.6 is set for Thole factors, which is actually the sum of atom-based Thole factors that default to 1.3 per atom. Electronic properties obtained from QM calculations such as dipole moments and molecular polarizabilities are usually targeted to calibrate these parameters. In contrast to the AMOEBA FF, an important feature of the Drude model is that atomic polarizability can be modeled via a diagonal rank-2 tensor with force constants that can be tuned to create anisotropic polarizabilities whenever a directional polarization response is required. This feature has been suggested to play an important role when modeling heterogeneous chemical environments^75^.

The Drude FF also makes use of virtual sites to model lone pairs on atoms that are hydrogen-bond acceptors and the *σ*-holes on halogens. As such, these lone pairs are positioned according to the ESP extracted from QM calculations and significantly improve the reproduction of quadrupole moments. Moreover, the use of lone pairs improves the directional response of hydrogen bonding, halogen bonding, and ion interactions^16,76,77^.

As with additive FFs, the Drude FF was developed after extensive and rigorous parametrization of small organic molecules^78–81^. The first Drude protein FF (called Drude-2013)^52^ was developed by deriving protein backbone and sidechain parameters, building upon previously parametrized model compounds. QM dipole moments, molecular polarizabilities, and water interactions of the alanine dipeptide and Ala_5_ were used as targets to develop nonbonded parameters for the protein backbone. After, extensive refinement of *ϕ*/*ψ* backbone dihedrals and *χ*_1_ and *χ*_2_ sidechain dihedrals based on QM data and empirical adjustments were carried out to improve agreement with PDB surveys. The Drude FF also uses the same CMAP approach used in the additive CHARMM FF to improve the agreement with *ϕ*/*ψ* distribution from PDB surveys. Simulations of peptides and folded proteins showed reasonable agreement with NMR data for a first-generation FF. The authors observed substantial variation in the dipole moment of amino acids in response to subtle changes in their microenvironment, an important feature for polarizable force fields. This observation was later emphasized by Huang et al. ^82^, who demonstrated profound sidechain dipole response of one glutamine residue as a function of its rotation in and out of a hydrophobic microenvironment in ubiquitin. Moreover, the authors showed that the first solvation shell around charged amino acids had larger dipole moments, which cannot be modeled using an additive FF and might be crucial for accurately capturing a proper balance between solute–solute and solute–solvent energies.

Later, Huang and MacKerell^83^ studied the helix formation of the (AAQAA)_3_ peptide using both Drude-2013 and CHARMM36 FFs in conjunction with Hamiltonian replica-exchange MD simulations. With the Drude FF, they were able to demonstrate that cooperativity in helix formation is driven by dipole moment enhancements in the peptide bonds involved in (*i*, *i* + 4) hydrogen bonding. The polarization response was directly tied to the capacity of the Drude-2013 FF to reproduce the helical content data of (AAQAA)_3_ peptide as a function of temperature, a property that CHARMM36 was unable to capture. More recently, Davidson et al. ^84^ used the Drude-2013 FF to study the forces stabilizing amyloidogenic fibrils such as the 42-residue alloform of the amyloid *β*-peptide (A*β*_42_) and the microtubule-associated protein tau. The authors showed that water molecules within the hydrophobic cores of the fibrils depolarize to maintain more favorable interactions with these microenvironments, and that buried polar residues have altered dipole moments, both of which may contribute to fibril stability.

After further investigation, Lin et al. ^85^ refined the nonbonded parameters of molecular ions used as the basis of sidechains of charged amino acids, correcting some overly favorable free energies of solvation. This work led to an extensive revision of Drude-2013, culminating in the Drude-2019 FF^53^. The authors refined atomic polarizabilities of sidechain carbons, which led to a refinement of backbone *ϕ*/*ψ* parameters and *χ*_1_/*χ*_2_ sidechain dihedral parameters. In addition, nonbonded interactions between charged residues were optimized against QM interaction energies and experimental osmotic pressures. Validation simulations were carried out for 19 protein/peptide structures on the microsecond timescale. The refinements made in Drude-2019 substantially improved the agreement with experimental J-coupling constants and *S*^2^ order parameters. Simulations of the potassium KcsA channel and the gramicidin A channel were also performed to demonstrate the capacity of Drude-2019 to accurately model membrane proteins. Overall, the Drude-2019 FF was described as a robust FF capable of dealing with proteins in many different chemical environments on the microsecond timescale.

## Experimental data availability

Developing a FF is a challenge that is often limited by the underlying physics of the proposed model and the availability of experimental data to be used as reference. As we have discussed above, these experimental data are crucial for FF refinement. Over the last 50 years, the continual increase in structural data has paved the way for many advances in FF development. However, FF development is still in need of more experimental data on the structure of highly flexible macromolecules and smaller-scale conformational properties such as sidechain preferences. An analogous situation exists for thermodynamic properties of charged chemical species, which are important for nonbonded parameter refinement. Here, we highlight some experimental data that could positively impact FF development efforts.

### Better thermodynamic data

Biomolecular systems are inherently heterogeneous in that they have to model e.g., the hydrophobic protein interior and polar solvent with equal accuracy. Thus, it is important to consider such heterogeneity in parameter development. Doing so thus requires the consideration of interactions among different species, and calibrating parameters against water or assuming a uniform high dielectric medium may be insufficient.

While early FFs derived partial charges targeting QM calculations directly, the next generation of FFs included QM interaction energies as targets to refine interactions among biomolecular moieties, such as charged sidechain analogs and ions^5,6,13,14^. Although important, such interactions are computed in the gas phase and their applicability to the actual screened interactions in an aqueous solution is difficult to assess due to the difference in polarization response in each case. In contrast, some FFs targeted condensed-phase data directly such as liquid density (*ρ*_*l*_) and heat of vaporization (Δ*H*_vap_), like the original OPLS parametrization scheme by Jorgensen et al. ^15^. Other force fields also included free energy of solvation or hydration in their calibration pipeline, like the GROMOS 53A5/53A6 FFs developed by Oostenbrink et al. ^22^. By targeting Δ*H*_vap_, the authors assumed that a single set parameter could accurately reproduce the molecular behavior in two different polarization conditions (gas and condensed phase), which could bias the derived parameters. A similar assumption is made when targeting free energy of hydration or solvation^86,87^.

Lately, attempts have been made to rebalance interaction energies by targeting solution data^88^. Luo and Roux developed an MD protocol to calculate osmotic coefficients, which was applied to refine CHARMM FF parameters for Na^+^, K^+^, and Cl^-^ to reproduce experimental osmotic pressure coefficients at high salt concentrations, a challenging task for any additive FF. Later, Lay et al. ^89^ applied the same method to optimize solute–solute interactions of carbohydrates in both CHARMM and AMBER FFs. Miller et al. ^90^ assessed the AMBER99SB-ILDN force field in reproducing experimental osmotic pressure coefficients. The authors reported that intermolecular interactions were systematically too favorable when combining the AMBER FF with the commonly used TIP3P water model, which was not seen when using TIP4P-Ew or TIP4P-D water models. Such results suggest that a comprehensive benchmark of modern FFs against osmotic coefficients could further improve the solute–solute and solute–solvent interactions At the same time, such an undertaking requires a comprehensive repository of experimental osmotic pressure coefficients for protein backbone and sidechain analogs, which simply does not exist. Most of the available experimental data are decentralized and/or were obtained more than 50 years ago with methodological details that are sparse in comparison to modern publications^91–95^, which hampers all FF development efforts on this front. Obtaining such experimental data for multiple protein functional groups and making them publicly available would represent a critical step for the improvement of current FFs, allowing the new generation of both polarizable and additive FFs.

Using a similar concept, the Open Force Field Consortium has recently trained LJ parameters against properties of mixtures such as partial densities (*ρ*_*l*_(*x*)) and enthalpies of mixing (Δ*H*_*mix*_)^96^ obtained from the NIST ThermoML archive^97^. This approach was used to optimize the OpenFF 1.0.0 (“Parsley”) drug-like molecule FF^98^, which ultimately led to the development of OpenFFF 2.0.0 (“Sage”) FF^99^. By using properties of condensed-phase mixtures, the authors were able to better reproduce QM geometries and energies. Although focused on small molecules, a similar approach could be applied to amino acid analogs in order to refine solute–solute and solute–solvent interactions.

Overall, the incorporation of solution data of protein analogs could represent an important step to further improve solute–solute and solute–solvent interaction energies for both polarizable and nonpolarizable FFs, allowing to better capture protein structure, dynamics, and thermodynamics in the condensed phase.

### More diverse structural data

Historically, force fields aimed to stabilize the tertiary folds of proteins, relying heavily on PDB structures and survey data to define allowable conformational states. However, solution data have been a powerful complement to static structures, helping researchers determine the limitations of FFs and serving as new structural data for FF refinement^100–104^. Moreover, solution data have the benefit of incorporating conformational ensemble information of highly flexible macromolecules such as IDPs, short peptides, or glycosylated proteins into the FF development pipeline. The use of more diverse data sets simultaneously to perform FF parameter fitting should be emphasized going forward.

For instance, NMR data of the Ala_5_ peptide has been used to refine backbone parameters for nearly 20 years and many FFs parameters were validated using J-coupling data, residual dipolar couplings (RDCs), and S^2^ order parameters of entire proteins. Going beyond validation, some FFs used NMR data of proteins to refine parameters^6,13,30,105^ without losing agreement with X-ray structural data. More recently, Kümmerer et al. ^106^ developed an approach that fits MD trajectories into sidechain NMR relaxation measurements, highlighting areas in which FFs require further refinement. While modern FFs do provide good agreement with NMR data, the overlap of protein targets used in validations is large, and a broader set of experimental NMR target data might be necessary to test and refine FFs even further.

Repositories of solution data, such as the Biological Magnetic Resonance Data Bank (BMRMB, https://bmrb.io/)^107^, the Small Angle Scattering Biological Data Bank (SASBDB, https://www.sasbdb.org/)^108,109^, and the Protein Circular Dichroism Data Bank (PCDBB, https://pcddb.cryst.bbk.ac.uk/)^110,111^ play an important role on protein FF development studies. The SASBDB has been growing steadily since its release in 2014. The radius of gyration values derived from SAXS data have been used in the development of CHARMM36m, ff99SB-disp, and ESFF1 FFs while specifically targeting IDPs. A similar approach has been used by incorporating NMR and circular dichroism spectral data in the FF development pipeline. Nevertheless, using a broader range of experimental outcomes to refine and validate FFs can be useful in judging the quality of the conformational ensemble produced.

Overall, the use of solution data has been pivotal to the development of more robust models in the last decade, and the influence and importance of such data are expected to grow as the availability of more comprehensive data sets are made public.

## Challenges and opportunities in parameter fitting

While early FFs suffered from the lack of computational power at the time to validate their parameters by simulating large protein target sets on the microsecond timescale, current challenges are more directly related to the amount of available training data and possible biases when refining parameters.

To overcome implicit biases, machine learning (ML) methods are emerging as a powerful tool to reduce much of the empirical tweaking often involved in FF development, yielding more robust parameter sets in a more efficient manner.

Although no protein FF has yet been developed using ML approaches, some promising cases should be mentioned. The Roitberg group developed the so-called ANI approach, a deep neural network trained on QM DFT data, to derive parameters for small organic molecules with remarkable accuracy^112,113^. The authors concluded that the most recent model, ANI-2x, produces 1D and 2D torsional energy profiles with similar accuracy as *ω*B97X/6-31G* calculations and outperforms MMFF94 and OPLS3 force fields. Nevertheless, the ANI approach was developed with the aim of developing parameters for small molecules and its application to complex biomolecules such as proteins has yet to be demonstrated. On a similar path, the Open Force Field (OpenFF) initiative has been consistently and successfully applying ML approaches as their core method to derive parameters for small organic molecules^98,99^. After the release of their newest small molecule parameter set, OpenFF introduced in their roadmap (https://openforcefield.org/about/roadmap/) a goal to move toward biomolecules in the near future^114^.

More recently, the CHARMM36 lipid force field^100^ employed a semi-automatic approach to fit bonded and nonbonded parameters of lipid head groups altogether. The authors used perturbed parameters in high-dimensional space and applied to reweight procedures to determine optimal solutions. In terms of polarizable FFs, the MacKerell group employed neural networks in the development of electrostatic parameters for small organic molecules based on the Drude polarizable FF^115^. The authors were able to determine atomic polarizabilities and partial charges in excellent agreement with high-level QM data, even though just information regarding molecular connectivity was parsed to the algorithm. Such findings suggest that both molecular polarizability and partial charges can be well described by additive contributions of proximal atoms, decreasing the workload usually employed when deriving electrostatic parameters.

Ultimately, FF parametrization is a problem that must be solved in high-dimensional space, and, historically, the interdependence of each parameter makes it difficult to isolate them for the purpose of the fitting. However, methods like the ForceBalance algorithm^32^ and the ones described above can be seen as demonstrations that automated parameter fitting procedures are powerful and can reduce implicit bias and overfitting while refining multiple parameters at once. In this sense, the growing use of such methods suggests that the quality and availability of the required experimental data targeted could soon become the main bottleneck in FF development.

An interesting demonstration of how powerful the combination of empirical biophysical data and machine learning approaches can be the work of Tesei et al. ^116^. The authors used a coarse-grained model along with a modified LJ expression that takes into account a residue-specific hydrophobicity parameter (*λ*) that modulates the original LJ energy term. The authors trained *λ* values using SAXS and paramagnetic relaxation enhancement NMR data of 45 IDPs selected from the literature through a series of state-of-the-art simulations, which allowed them to reproduce sequence-specific propensities of IDPs to undergo liquid-liquid phase separation and form condensates. Although more testing is needed to demonstrate the general applicability of this model for IDPs not used in the training phase, the authors envision the refinement of their automated method as more experimental data are made available and even extend it for specific pairwise interactions such as cation-*π* interactions or post-translational modifications.

Such examples are clear demonstrations of how powerful automated fitting methods are when combined with comprehensive experimental data in refining FF parameters to avoid overfitting issues or unconscious biases. Further improvements and applications of such methods in FF development are expected to have a profound impact on our field.

## Conclusions and outlook

Although many innovative methods have been applied to further improve these models, the underlying physics applied to additive FFs throughout the last 20 years is known to only achieve some quantitative accuracy due to their limited representation of inter- and intramolecular energies. Even though polarizable FFs are very recent and might still need thorough usage and testing, they demonstrate great potential in better reproduction of inter- and intramolecular energies of proteins in different chemical environments. The recent advances in computing hardware and software allow polarizable MD simulations to reach the microsecond timescale in a feasible timeframe, making these robust models more available.

Nevertheless, both additive and polarizable FFs will benefit from the availability of comprehensive experimental data. The increased incorporation of solution data from ever-diverse sources and the inclusion of ML approaches to derive more robust parameters will play an integral role in FF refinement and ultimately the quality of MD simulations arising from their application. To do so requires access to high-quality structural and thermodynamic data, ideally in machine-readable formats and residing in open repositories. Moving forward, objective fitting functions and other ML approaches will rely on these data to improve existing FFs and derive new ones. In this regard, the theoretical and experimental communities will equally benefit from more robust models and associated empirical data.
